# A portable organic plastic scintillator dosimetry system for low energy X-rays: A feasibility study using an intraoperative X-ray unit as the radiation source

**DOI:** 10.4103/0971-6203.33245

**Published:** 2007

**Authors:** Kerry Williams, Neil Robinson, Jamie Trapp, Trevor Ackerly, Ram Das, Penny Kemp, Moshi Geso

**Affiliations:** School of Applied Sciences, RMIT University, Melbourne, Victoria 3000; * Department of Physical Sciences, Peter MacCallum Cancer Institute, East Melbourne, Victoria 3001, Australia

**Keywords:** Dosimeter, low energy intraoperative radiotherapy organic plastic scintillator

## Abstract

The effective use of near water equivalent organic plastic scintillators (OPS) for radiation dosimetry with high-energy sources under laboratory conditions is recognized. In this work, an OPS-based dosimeter using a photodiode combined with improved solid state detection and signal processing techniques has been developed; it offers the potential for the construction of a stable and fully portable dosimeter which will extend the useful range of measurement beyond the usual MeV area and provide reliable readings down to sub-‘100 keV’ X-ray energy levels. In these experiments, the instrument described has been used for the dosimetry of INTRABEAM intraoperative radiotherapy (IORT) equipment at distances as low as 1.8 mm from the effective source, i.e., 0.2 mm from the X-ray probe surface. Comparison is shown with dosimetry measurements made using the calibrated reference ion chamber supplied by the IORT equipment manufacturer.

Radiation dosimetry procedures for intraoperative radiotherapy[1] (IORT) equipment are often performed with parallel plate ionization chamber type dosimeter, a device based on the principle of charges collected due to ionization within their sensitive air volume.[2] The main condition imposed on their satisfactory performance is the fulfillment of charge equilibrium.[2,3] Thus reliable measurement close to the source can prove problematic. System manufacturer's data is provided only for distances of 5 mm and greater from the X-ray source.[4,5] Dose measurements as close as 3 mm from the IORT source using a specially modified ion chamber[6] have been presented by Beatty *et al.* and by Ebert and Carruthers;[7] however, when measuring dose rates under clinical conditions using an ‘ion chamber’-based dosimeter, readings may become unreliable as one approaches the near field due to the steep gradients encountered, the critical geometry of the chamber volume and field perturbation effects caused by its presence. The use of an organic plastic scintillator as the radiation detector can overcome these limitations and provide a seamless sequence of readings up to the surface of the IORT probe.

Detectors utilizing organic plastic scintillators are largely immune to environmental variables and also offer the advantage that they do not saturate at high dose rates. OPS offer the advantages of near water equivalence and offer minimal disturbance of the radiation field; they are not influenced by changes in ambient conditions of temperature and pressure, are uncritical as to angle of incidence of radiation[8,9] and can provide an immediate direct reading. OPS detectors may be machined to very small dimensions of only a few cubic millimeters; yet they offer a wide dynamic response range with high spatial resolution and remain effective at the high dose rates encountered close to the radiation source. Organic plastic scintillators have previously been successfully tested as dosimeters for high-energy radiation, at MeV energies.[9] Details of the IORT probe and dosimetry techniques used in its clinical certification are described by Beatty;[6] they show the position of the gold target within the probe housing and also provide an explanation of the method used to achieve a virtual point source radiator.

The experimental setup of the PRS400 X-ray probe, water phantom and the OPS dosimeter, as depicted in Figure 1, shows the basic geometry of the system. With the OPS probe in contact with the beryllium window, the surface of the scintillation material will be ~1.8 mm from the effective source position within the IORT source probe housing. The OPS detector element fabricated from BICRON^®^ BC-430 scintillator material forms the active tip of a probe. It consists of a disk of this material 4.5 mm in diameter and 2.5 mm thick. A 2 mm diameter light guide (BICRON^®^ BC-98) 800 mm long is bonded to a recess machined in the edge of the scintillator disk as shown in Figure 1. The surface of the probe is covered with a black acrylic coating to inhibit the entry of external light and to provide waterproofing.

**Figure 1:**
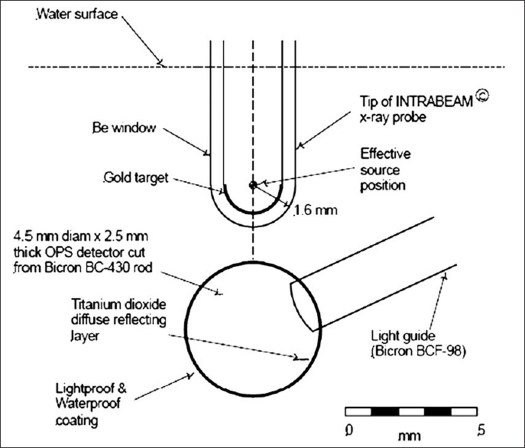
Arrangement of components showing tip of the X-ray probe (PRS400 system) and OPS detector. The scintillator element, constructed from BICRON BC-430 plastic organic scintillator, is a disk 4.5 mm in diameter and 2.5 mm thick. The scintillator is coated with a diffuse reflecting layer of titanium dioxide and a lightproof and waterproof acrylic coating to protect the probe during immersion in water. The light guide is 2 mm diameter BICRON BCF-98 (clear plastic) bonded to a machined recess on the disk circumference using BICRON BC-600 adhesive; it couples the scintillator element to a photodiode mounted in a remote shielded steel housing. All measurements were taken with the X-ray source probe and scintillator immersed in water

The remote end of the plastic light-guide, coated with optical grease (BICRON^®^ BC-630), is clamped to the window of a UDT Sensors^®^ 5DPI photodiode, which constitutes the input device for the signal processing circuitry and data acquisition system. Photons enter the window of the photodiode and induce a minute electron current in the analogue buffer stage. The output of the buffer is connected directly to a dedicated analogue-to-digital (A/D) converter operating in the nano-amp region. The A/D conversion concept was developed around the LTC 1799 integrated circuit configured as a current-to-frequency converter and is used to generate a pulse train at TTL level, the frequency of which is proportional to the output signal level from the photodiode. The resulting signal is buffered and transferred to a personal computer via a USB-linked data acquisition system for storage, analysis and display. An overview of the electronic signal processing system and circuit functions is given in Figure 2A, and a typical data stream from the instrument is shown in Figure 2B.

**Figure 2A:**
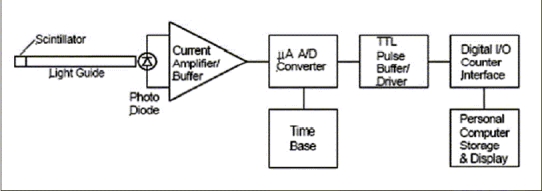
Block diagram showing the configuration of the dosimeter used in these experiments. Light from the OPS is coupled to a photodiode and current buffer/amplifier. A current-to-frequency conversion takes place in the following stage, where a precision current-controlled oscillator generates the base or ‘dark’ frequency. Under radiation, the system produces a frequency shift proportional to beam intensity. The resulting signal is conditioned and buffered for TTL compliance and then passed to a digital I/O interface connected to a personal computer for data storage and display

**Figure 2B:**
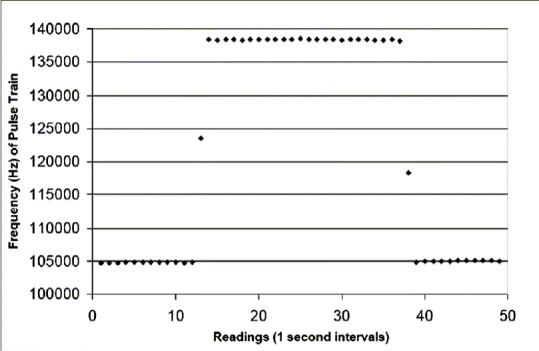
Contents of a typical data file of one radiation event (beam on between 12 and 39 s). Spot readings are taken and stored at one-second intervals over a period sufficiently long as to provide acceptable signal averaging

Performance and linearity of the system were checked against the PTW Type N23342 parallel plate ion chamber reference supplied with the IORT equipment. The system was tested at various beam currents at a fixed distance from the source in air, and a further series of measurements was made at a fixed beam voltage and current in the water tank at varying distances from the source. For these tests, the probe element was attached to the outer surface of a plastic (solid water) holder housing the parallel plate ionization chamber. The probe disk was aligned edge-on to the radiation source and the complete assembly immersed in the water tank. The position of the OPS detector and ion chamber relative to the surface of the radiation source was adjusted in the water tank by use of external control arms that allow movement in three planes to an accuracy of ±0.25 mm.

## Results and Discussion

**Figure 3:**
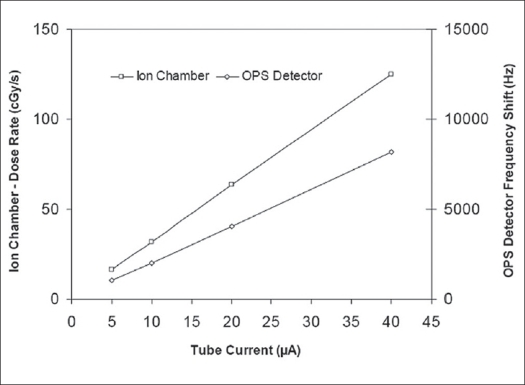
Output of OPS dosimeter and ion chamber readings at 2 mm distance vs. X-ray tube current showing linearity of detector at a fixed energy of 50 kVp. Note 1 - Uncertainty limits (recorded in Table 1) are too small to show graphically. Note 2 - The slope of the curves is a function of the scales chosen for purposes of clarity and is not an indication of relative sensitivity of the detectors

Prior to carrying out the series of measurements in the water tank, the apparatus was ‘bench-tested’ at 50 kVp, which is the set energy level used in the clinical situation. Ion chamber readings and OPS dosimeter measurements at a distance of 2 mm from the source surface (in air) were logged and compared over the available range of tube currents - from 5 μA to 40 μA. Numerical data showing the dose measured with the ion chamber and the equivalent output reading from the OPS dosimeter are shown in Table 1. A graphical presentation of the OPS data from Table 1 is shown in Figure 3, together with dose readings from the ion chamber reference. A calibration factor for the particular configuration can be calculated from the data, which will allow interpolation of readings across the energy range of interest. When carrying out measurements over a broad range of X-ray energies or when physically changing the OPS probe assembly, a new calibration factor for the particular probe configuration would need to be established and entered into the data processing function.

**Tabel 1:** Organic plastic scintillators (OPS) detector and ion chamber readings at 2 mm from radiation source in air. The level of uncertainty in the OPS detector outputs is quoted at 95% confidence limits. Column 2 shows output frequency of the OPS instrument, and column 3 shows frequency shift from the dark (no radiation) state

*X-ray tube current (μA)*	*OPS detector reading (kHz)*	*OPS detector frequency shift (Hz)*	*Dose rate (Ion chamber) (cGy/s)*
0	95.758		
5	96.808	1050±36	16.15
10	97.756	1998±33	32.0
20	99.787	4029±33	63.5
40	103.953	8195±31	125.0

OPS - Organic plastic scintillators

**Figure 4:**
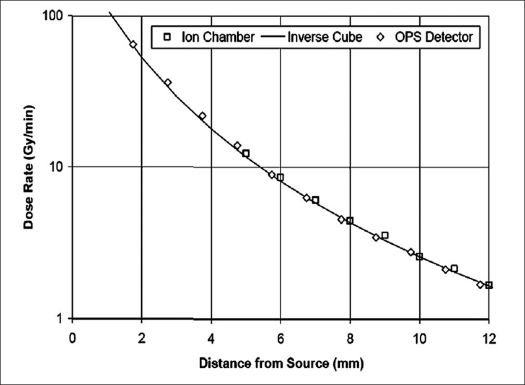
Dose rate vs. distance in water for ion chamber and OPS detector. Note close agreement with inverse cube function

A further series of measurements were taken in the water tank, and the results are shown in Table 2. This data is presented graphically in Figure 4. Note that the closest distance from the active area of the plastic scintillator to the surface of the source window is limited by the thickness of the titanium dioxide layer as also by the light blocking the plastic film covering the scintillator. The total thickness of the two layers is approximately 0.2 mm.

**Table 2:** Ion chamber and organic plastic scintillators detector readings from source surface to 16 mm distance in water

*Distance from source (mm)*	*Dose rate-Ion chamber (Gy/min)*	*Dose rate-OPS detector (Gy/min)*
1.8		67.74±0.09 → 0.1%
2.8		36.41±0.09 → 0.2%
3.8		21.98±0.11 → 0.5%
4.8		13.98±0.09 → 0.6%
5.0	12.39	
5.8		8.96±0.11 → 1.2%
6.0	8.60	
6.8		6.33±0.11 → 1.7%
7.0	6.12	
7.8		4.56±0.13 → 2.9%
8.0	4.45	
8.8		3.47±0.09 → 2.5%
9.0	3.58	
9.8		2.77±0.10 → 3.5%
10.0	2.59	
10.8		2.13±0.12 → 5.4%
11.0	2.16	
11.8		1.70±0.11 → 6.2%
12.0	1.67	
13.0	1.36	
14.0	1.14	
15.0	0.96	
16.0	0.80	
16.8		0.71±0.11 → 15.4%

Note that due to the small volume of scintillator used (40 mm^3^), percentage uncertainties rise signi.cantly beyond 12 mm.

The measurements extend from this lower limit out to a distance of 16 mm from the source. Beyond this point when using the selected probe of volume 40 mm^3^, OPS dosimeter readings progressively fall to a level where due to the small volume of the scintillator element, background noise levels become significant.

Dose measurements previously made using a liquid water phantom by Beatty *et al.* were found to closely approximate an inverse cube function with distance.[6] A confirmation of this characteristic can be observed in the output of the OPS dosimeter plotted in Figure 4, which shows a plot of the dose rates measured by the OPS detector and ion chamber for distances up to 12 mm from the source surface. Across this range, the plot indicates very close agreement between the OPS measurements and the reference ion chamber readings. The resulting curves have been overlaid such that the correct relationship to the effective source position is maintained and additional measurements taken with the OPS dosimeter extend the readings down to the minimum possible distance of ~ 0.2 mm from the surface of the source probe. It can be seen that the curve continues to describe an inverse cube function, as would be expected.

Beyond 15 mm from the source, due to the relatively small volume of scintillator element used, the falling signal-to-noise ratio begins to limit resolution. Since dose rate measurement at these distances can effectively be covered using the ionization chamber method, the graphing of OPS dosimeter readings was limited in these tests to distances closer than 12 mm from the source and shows monotonic increase in dose rate up to about 65 Gy/min at the closest distance of approximately 0.2 mm from the external surface of the INTRABEAM X-ray probe.

## Conclusion

X-radiation emission from the PRS 400 intraoperative unit was measured using an organic plastic scintillator dosimeter over distances ranging from 0.2 mm to beyond 12 mm from the source surface. IORT probe manufacturer's data, measured using an ion chamber dosimeter, is provided for distances of 5 mm and greater from the X-ray source. The use of the instrument outlined in this work offers the possibility of another method of dosimetry for the PRS400 system and also extends the range of available dose rate readings down to distances of less than 5 mm from the source. The advantageous characteristics of OPS detectors allow reliable measurements to be made right up to the radiation window, where steep dose gradients and the beam-distorting effects of other types of dosimeters render results questionable. The facility to achieve reliable near water equivalent dose measurements at close distances from the radiation source using a portable detection instrument that is simple to apply and operate could be of considerable interest to users of IORT systems.
